# The role of torso stiffness and prediction in the biomechanics of anxiety: a narrative review

**DOI:** 10.3389/fspor.2024.1487862

**Published:** 2024-11-01

**Authors:** Seong Chin

**Affiliations:** Department of Medicine, Advocate Lutheran General Hospital, Park Ridge, IL, United States

**Keywords:** anxiety, torso stiffness, prediction, venous return, intra-abdominal pressure, aerobic exercise, autism

## Abstract

Although anxiety is a common psychological condition, its symptoms are related to a cardiopulmonary strain which can cause palpitation, dyspnea, dizziness, and syncope. Severe anxiety can be disabling and lead to cardiac events such as those seen in Takotsubo cardiomyopathy. Since torso stiffness is a stress response to unpredictable situations or unexpected outcomes, studying the biomechanics behind it may provide a better understanding of the pathophysiology of anxiety on circulation, especially on venous impedance. Any degree of torso stiffness related to anxiety would limit venous return, which in turn drops cardiac output because the heart can pump only what it receives. Various methods and habits used to relieve stress seem to reduce torso stiffness. Humans are large obligatory bipedal upright primates and thus need to use the torso carefully for smooth upright activities with an accurate prediction. The upright nature of human activity itself seems to contribute to anxiety due to the needed torso stiffness using the very unstable spine. Proper planning of actions with an accurate prediction of outcomes of self and non-self would be critical to achieving motor control and ventilation in bipedal activities. Many conditions linked to prediction errors are likely to cause various degrees of torso stiffness due to incomplete learning and unsatisfactory execution of actions, which will ultimately contribute to anxiety. Modifying environmental factors to improve predictability seems to be an important step in treating anxiety. The benefit of playful aerobic activity and proper breathing on anxiety may be from the modulation of torso stiffness and enhancement of central circulation resulting in prevention of the negative effect on the cardiopulmonary system.

## 1 Introduction

Anxiety can be a normal presence in stressful situations. Still, excessive anxiety can lead to a panic attack, which can cause the sensation of suffocation and doom, as well as common symptoms of palpitation, rapid breathing, sweating, and dizziness. Some people may pass out, risking injuries. Anxiety is also linked to an increased risk of cardiovascular disorders (1), premature mortality (2), sudden cardiac arrest (3), self-harm (4, 5), and suicidality that is not explained by depression (6, 7). While the pathophysiology of anxiety and panic disorder is not well understood, many professionals advise doing other things to take one's mind off of the stressor: knitting, coloring books, deep breathing, regular exercise, hiking, and partaking in hobbies.

Hyperventilation is common in panic attacks and characterized by a shift from a diaphragmatic to a thoracic breathing pattern, which imposes a biomechanical stress on the neck and shoulder region due to the ancillary recruitment of the sternocleidomastoid, scalene, and trapezius muscles in support of thoracic breathing (8). In hyperventilation theory, hyperventilation causes a drop in arterial CO_2_ and a change in the blood pH (i.e., respiratory alkalosis), disrupting blood acid-base equilibrium. This disruption can further induce adverse effects, including muscle tension and muscle spasms from an amplified response to catecholamines. Even though hyperventilation theory is appealing and provides some clues to many symptoms of anxiety and panic disorders, this theory has lost popularity due to a lack of experimental evidence (9).

Instead, aerobic exercise, which would cause hyperventilation, appears beneficial in mitigating anxiety (10). Even acute aerobic exercise mitigates negative symptoms of obsessive-compulsive disorder (OCD), a severe form of anxiety disorder characterized by unwanted thoughts and repetitive irrational behaviors (11). While the beneficial effect of physical activity on anxiety seems to be far superior to medications and cognitive behavioral therapy (12), there is no single major mechanism to explain the positive effects of exercise, thus limiting the development of optimal strategies in the application of exercise for the treatment of anxiety (10, 13).

Anxiety with an increased adrenergic tone has a lowered heart rate variability (HRV) (14) which is a valuable tool to evaluate the function of the autonomic nervous system. HRV is lower in cardiovascular disorders (15), metabolic disorders (16), and other mental conditions known to cause anxiety (17, 18). How aerobic exercise (19, 20) and quiet breathing (21) improve HRV may be understood through a biomechanical approach to the effects of anxiety on circulation and ventilation.

In this review of the biomechanics of anxiety, we will discuss the complexity of bipedalism which is the evolutionary hallmark of humans for energy-efficient endurance running and walking (22). Using the upper body and arms via successful bipedalism, humans developed sophisticated skills to use various tools including weapons. However, due to inherent instability, upper body activity requires torso stabilization and accurate prediction for balance in complex bipedal locomotion. Torso stiffness (23) is common in anxiety and seems to present as various symptoms of the circulatory system. A biomechanical approach to the roles of the respiratory muscles and abdominal wall muscles in torso stabilization (24–28) and circulation (29–33) may help understand the pathophysiology of anxiety in many disorders including autism.

## 2 Expanded spinal canal and trunk control in bipedal humans

In a study on the evolution of human speech (34), the authors suggested that the evolution of breathing control through increased thoracic innervation to abdominal muscles and thoracic muscles (including intercostal and subcostal muscles) was essential for developing human language. They reported that Neanderthals and early modern humans had expanded thoracic canals. They also suggested that earlier hominids did not possess the enhanced breathing control necessary for modern language until at least 1.6 Mya, the time of *Homo ergaster* (or early *Homo erectus*). Increased thoracic innervation to thoracic muscles and abdominal muscles is also necessary for many functions of humans: upright bipedal locomotion, flexion, breathing, and expulsion (e.g., coughing and defecation).

Compared to Eocene primates, the evolutionary expansion of the spinal canal in modern-day primates is to support sophisticated locomotor control of both the forelimb and the hindlimb (35). Spinal cord expansion in early hominins occurred well before the expansion of the brain and was necessary for complex balance control of obligate bipedal movement (36). In addition, the control of breathing for speech needs delicate control of the thoracoabdominal muscles to prevent the fading of sound volume from the uncontrolled elastic recoil of the lungs. Humans can produce a more than 7-fold increase in the duration of exhalation compared to the 2–3 times increase seen in the majority of nonhuman primate species (34).

## 3 Functional anatomy of torso stabilization

### 3.1 Abdominal wall muscles

Among the multiple anterior abdominal muscles, the rectus abdominis (RA) is a paired muscle located in the midline of the anterior wall. This muscle is covered by a rectus sheath which consists of the aponeuroses of the lateral abdominal muscles. The transverse tendinous intersections of the muscle give the appearance of a “six pack”. It runs vertically between the anterior lower rib cage and the pubic bone. It functions as an antagonist to the erector spinae muscles to flex the lumbar spine during sit-ups. It is also important for respiration during forced expiration. The sides of the abdominal wall are supported by the external oblique muscle (EO), the internal oblique muscle (IO), and the transversus abdominis muscle (TrA) (37).

The external oblique muscle (EO) is the most superficial of the three flat muscles of the lateral anterior abdomen. It is attached to the external surfaces and inferior borders of the fifth to twelfth ribs. The serratus anterior muscle is in proximity to the EO on the ribs. The aponeurosis of the external oblique muscle forms the inguinal ligament (37).

The internal oblique muscle (IO) is located just under EO and over TrA. It runs perpendicular to EO. Beginning in the lumbodorsal fascia of the lower back, IO reaches the anterior 2/3 of the iliac crest and the lateral half of the inguinal ligament. The muscle fibers run up to the inferior borders of the 10th through 12th ribs and the linea alba (abdominal midline seam). During expiration, it can contract to compress the abdominal cavity to push the internal organs up into the diaphragm while the diaphragm is returning back into the chest cavity with the elastic recoil of the lung structure (37).

The transverse abdominal muscle (TrA) is the innermost of the flat muscles of the abdomen just under IO. It has many attachment sites: the lumbodorsal fascia, the lateral third of the inguinal ligament, the anterior three-fourths of the inner lip of the iliac crest, the inner surfaces of the cartilages of the lower six ribs, and the diaphragm. It inserts into the linea alba; its upper three-fourths lie behind the rectus muscle and blend with the posterior lamella of the aponeurosis of the internal oblique; its lower fourth is in front of the rectus abdominis. The contraction of TrA can help to compress the ribs and viscera to provide spinal stability (37).

### 3.2 diaphragm and pelvic floor muscles in torso stabilization

In a study on diaphragm contraction (24), the authors found that the electromyogram (EMG) activity of the diaphragm and the transverse abdominal muscle (TrA) showed about 20 milli-seconds earlier to the onset of the EMG activity of the deltoid muscle during rapid flexion of the shoulder to a visual stimulus. The authors also visualized the eccentric contraction of the diaphragm after initial shortening (at the time of low intra-abdominal pressure) when TrA was co-contracting to increase the intra-abdominal pressure. The contraction was independent of the phase of respiration. However, during rapid movement of the thumb and wrist, a posture not nearly as challenging as the shoulder, was not associated with this anticipatory EMG in the diaphragm. The simultaneous contraction of the diaphragm and TrA was thought to be pre-programmed and not from reflex since the EMG activities preceded that of the deltoid muscle. It was also considered to contribute postural stability of the human trunk during sudden voluntary movement of the limbs.

In another study (25), the authors found that, during limb movement (flexion and extension of a shoulder joint), the diaphragm and TrA were tonically active with added phasic modulation at the frequencies of both respiration and limb movement. They also measured intra-abdominal pressure (measured through intragastric pressure: Pga), which increased during limb movement in proportion to the reactive forces from the movement. The rapid repetitive limb movement during breathing increased mean Pga by 26 cmH_2_O. During the repetitive limb movement, the diaphragm and the trunk muscles (TrA, EO, RA, and the erector spinae muscles/ES) were contracting tonically. The diaphragm and TrA showed varying patterns in EMG activity with respiration but the other trunk muscles (EO, RA, and ES) did not have the variation in EMG amplitude between respiratory phases. They concluded that the coactivation of the diaphragm and abdominal muscles caused a sustained increase in intra-abdominal pressure (Pga). They suggested that the increase of the Pga and the tonic activity of the diaphragm contributed to the mechanical stabilization of the trunk during the movement of limbs.

In a study (26) on the co-activation patterns of abdominal muscles in response to the contractions of pelvic floor muscles (PFM), the authors reported that the contraction of abdominal muscles was a normal response to PFM activity. In another study on the postural and respiratory functions of PFM (27), the EMG activity of PFM was increased in advance of deltoid muscle activity as a component of the pre-programmed anticipatory postural activity (as seen in the diaphragm and TrA). The EMG activity of PFM was primarily modulated in association with arm movement with little respiratory modulation. PFM with the diaphragm and other abdominal muscles seem to take an important role in generating and sustaining intra-abdominal pressure (IAP) to stabilize the spine for posture control. In a study using biomechanical models of the spine and its musculature, the researchers found that spinal compression force was lower, with an increase in IAP through the contraction of antagonistic muscles of the abdominal wall (28).

### 3.3 Other muscles in torso stabilization

Through the unique anatomical location and complex vertebral attachment (37), the psoas muscles (PS) can provide postural stability for the trunk (38) and spread the force across the lumbar area (39). However, there was no active EMG activity of PS during quiet sitting (38). The lack of PS activity during quiet sitting can have implications for the structural stability of the spine in the modern-day lifestyle with excessive sitting.

The erector spinae muscles (ES), which are important extensor muscles of the back, can be silent when the spine column is flexed beyond a certain angle, known as the flexion-relaxation phenomenon. Interestingly, the EMG activity of PS disappeared when ES were silent in a flexion position (40). At this point, force distribution for spinal stability via ES and PS would be very difficult. In a study on abdominal wall muscle activities during quiet breathing (reflecting non-stress conditions), the abdominal muscles had no muscle activity (41). Some apprehended participants had irregular activities (which ceased altogether later) of one or two abdominal muscles unrelated to the phases of respiration. The intragastric pressure (Pga) at the end of a quiet expiration was 1–15 cmH_2_O above atmospheric pressure (42) with the overall variation being no more than 3–5 cmH_2_O and there was no muscular activity of abdominal muscles during the quiet breathing. During maximal voluntary breathing, the Pga rose abruptly to over 50 cmH_2_O at the beginning of each expiration. This type of rise in the Pga during expiration never occurred in the absence of voluntarily controlled breathing even if the subjects became cyanosed and distressed in the involuntary respiration of asphyxia. In a study on maximal inspiratory and expiratory efforts (43), the Pga could rise to a range of 57–183 mmHg (77–250 cmH_2_O).

In a study on lumbopelvic stability and co-contraction of the lumbopelvic muscles (44), the authors found that, during the unpredictable trial of stability challenge, TrA remained active when the EMG activity of all lumbopelvic muscles was decreased. The EMG activity of TrA did not differ between the predictable and unpredictable trials. However, there was a tendency for TrA EMG activity to be greater when there was less predictability of the perturbation. During the lumbopelvic flexion and extension trials, TrA contraction (which does not have a directional torque when activated unlike other abdominal wall muscles) appeared to be more important for stabilization during the physical disturbances. TrA contraction, which stabilizes the torso and spine by raising IAP (45), can be important for posture control during responses to unpredictable situations or outcomes.

In another study on sudden perturbations on the trunk (46), TrA was always the first abdominal muscle (other muscles are EO/IO/RA) to be activated. The contraction of the abdominal muscles and diaphragm with an increase of IAP appears to cause increased stiffening of the intervertebral joints within the lumbar spine. Additionally, the authors noted that there was a rapid backward head movement during unexpected ventral loading. This extension movement of the head/torso seems to be a common reflexive motion that we make when we miss a step or encounter a surprise.

## 4 Guyton's venous return curve and intra-abdominal pressure

Bipedalism is the hallmark of human locomotive success and our ancestors were able to chase their prey through successful bipedalism and endurance running and walking (22), were able to use the arms, and developed language via delicate breathing control with complex thoracic innervation to the abdominal muscles and thoracic muscles (34). However, they still had to manage to stabilize the torso/pelvis during bipedal running on uneven natural surfaces due to the inherently unstable posture during running, as the ligamentous spine without muscular contribution is very unstable (47, 48). For the upright posture and applied stiffness to the trunk for various upper body activities, a degree of trunk stiffness is expected (25). This stiffness will affect venous return negatively, considering the very low central venous pressure (3–8 mmHg), right atrial pressure (2–6 mmHg), and left atrial pressure (6–12 mmHg). If prolonged venous impedance continues during static upright upper body activities without proper rest, extrinsic impedance will decrease the cardiac preload and performance.

Considering Guyton's venous return curve (49) with a basic principle that the heart can pump out only what it receives from the large reservoir of circulatory volume (50), the proper venous return seems critical for proper cardiac function and tissue perfusion. In the equation of the venous return curve in steady conditions, he showed that the right atrial pressure and cardiac output are inversely related (14% drop in cardiac output by every 1 mmHg increment in pressure). While the implication of his findings is very important in caring for patients, many agreements (51) and disagreements (52) surround the equation from the lack of agreement on the hypothetical mean circulatory filling pressure (MCFP, the average integrated pressure throughout the circulatory system) which needs to be higher than the right atrial filling pressure.

Considering the very high intra-abdominal pressure (IAP) of a range of 57–183 mmHg (77–250 cmH_2_O) during maximal respiratory efforts, although very low IAP is achievable with a little variation during quiet breathing (42, 43), it is evident that there will be a negative impact on circulation by venous impedance (53) if a degree of torso stiffness during strenuous or stressful upright bipedal activities is prolonged. Increased adrenergic tone not only causes excessive shortening of cardiac sarcomeres (contraction band/necrosis) (54) but also worsens torso stiffness in response to decreased preload if not reversed fast enough. This contraction band necrosis of cardiac muscles is found in stressed animals (55) and humans (56). To become successful hunter-gatherers, human ancestors with endurance running must have achieved proper motor skills for safe and proper venous return. To summarize, excessive torso stiffness, if not handled safely, can deteriorate venous return and be destructive to myocardial structural integrity causing myocardial rigors.

A pressure higher than 10 mmHg in the peritoneum is defined as “Intra-Abdominal Hypertension” which needs to be avoided (57). The negative effect can be observed even at 8 mmHg in animal models (58). The increase in muscular stiffness and IAP will contribute to an increase in intrathoracic pressure and a decrease in cardiac output. In a study on the relationship between abdominal pressure, pulmonary compliance, and cardiac preload in a porcine model (59), the authors found that increased IAP was transferred to the thoracic compartment and resulted in decreased respiratory system compliance (due to decreased chest wall compliance) and stroke index (SI: ml/kg). They noted a 22% decrease in SI at 30 mmHg of IAP. Filling pressures like CVP (central venous pressure) and PAOP (pulmonary arterial occlusion pressure) also increased significantly in response to increasing IAP which indicates the increased resistance to filling cardiac chambers and subsequent drop of cardiac stroke volume.

While proper weight-bearing activity (60) and quiet breathing (61) are the most important mechanisms for venous return in humans, prolonged static sitting causes decreased venous return and cardiac output with a compensatory increase in adrenergic tone. The lack of contribution from the PS during quiet sitting (38) and the inherent instability of lumbar spines (48) will require activation of TrA and other abdominal wall muscles (62) with related increases in IAP accordingly. Various degrees of stress response (63) and stiffening of the abdominal wall muscles (25) are expected depending on the level of risk involved in the given tasks. Seemingly harmless quiet sitting with a hydrostatic pressure of 28–32 mmHg in the pelvis (64) can reach a harmful level of IAP in the pelvis during a prolonged sitting to cause various negative impacts (57, 58, 65) which would get worse during demanding near-point work on computer screens (66). When venous return gets limited during sitting by the inactive skeletal muscles and restricted respiratory muscles, additional hydrostatic intraabdominal pressure can harm internal organs, and even worse if related risk is high.

## 5 Expiratory flow limitation and dynamic hyperinflation: biomechanics of dyspnea

Dyspnea is a common symptom of anxiety (67) and people with panic attacks report that they are very frightened by the feeling, sometimes referencing feelings of doom and a feeling that they are going to die from the sensation of choking. The mechanisms and pathways of the sensation of dyspnea are not completely understood (68). Juxtacapillary (J) receptors (or pulmonary C-fiber receptors) are non-myelinated slow-acting sensory vagus nerve fibers located within the alveolar walls in juxtaposition to the pulmonary capillaries of the lung. They are believed to be “nociceptors” responding to tissue damage and edema. They are stimulated during pulmonary congestion produced by occluding the aorta or left atrioventricular junction, which causes an increase in left atrial pressure with a consequent rise in pulmonary arterial pressure.

In feline experiments (69), there was a delayed response time (5–10 s) after the start of the occlusion of the aorta or atrioventricular junction. The interval between the start of the rise in systolic right ventricular pressure and the onset of stimulation in different fibers varied considerably. In some fibers, it was as little as 2 s; in others, about 15 s or more. Two nerve endings were not excited even 30 s after occlusion occurred. This considerable lag was the same after an injection of alloxan (a stimulant used in similar experiments by others). The author believed that the main physiological stimulus to the receptors was an increase in interstitial fluid in the alveolar wall.

It appears that the activation of pulmonary C-fiber receptors in animals produces inhibition of spinal motoneurons, reflex bradycardia, apnea, and hypotension via the vagus nerve. The injection of lobeline (a drug that activates pulmonary C-fiber receptors) can cause small animals (like cats) to collapse from reflex spinal motor neuron inhibition. When lobeline is injected into human subjects, they report noxious sensations of smoke or fumes in their airways. The sensation evokes an element of fear. It contributes to the sense of dyspnea. Even though human subjects do not collapse after the injection, they develop bradycardia (a decrease of 10–15 beats/min) with the accompanying hypotension (a decrease of ∼40 mmHg). In human subjects, the inhibition at a spinal level by lobeline was thought to be overridden by increased descending excitation, either from arousal or voluntary cerebral command (70).

However, in the study on the mechanisms of stimulation of pulmonary C-fiber receptors (69), the author noted that the application of local pressure on the lungs of cats was a very effective way to localize the nerve endings. The peak frequency in response to pressure stimulation was greater than that following phenyl diguanide injection. He wrote:

“***Effects of local pressure.*** *Application of local pressure on the lungs was an effective method of stimulating the endings so that it became possible to localize the endings in the individual lobes… Some endings could be stimulated by stroking the surface of the lungs; others needed strong pressure in order to stimulate them… [It] was found that mechanical stimuli set off a relatively prolonged discharge of impulses which continued for several sec after the mechanical stimulus had been withdrawn.”*

From the observation above, we can infer that the most important function of pulmonary C-fiber receptors may be intrathoracic pressure monitoring during trunk control. This is essential for human bipedal locomotion and speech, especially with very low central venous pressure (3–8 mmHg), right atrial pressure (2–6 mmHg), and pulmonary capillary pressure (4–12 mmHg). Because human ancestors spent a significant amount of time as arboreal animals before becoming an obligatory bipedal species (71), they must have developed the necessary skills of trunk control which eventually became an essential part of bipedal locomotion and energy efficiency. Unlike quadrupedal animals, our human ancestors had to overcome the dilemma of controlling the needed stiffness of the trunk while avoiding excessive pressure on internal organs and vascular structures. Having a well-developed sensory system to monitor internal pressure can be accomplished via pulmonary C-fiber receptors. The varying degrees of response rate can be interpreted as the results of different degrees of applied pressure during activities of torso stiffness.

In a study (72) on the central integration of signals from pulmonary vagal C-fiber receptors along with those arising from cardiac, peripheral chemoreceptor, and baroreceptor afferents to neurons within the nucleus of the solitary tract, the author found that, after stimulation of pulmonary C-fiber receptors with phenylbiguanide, none of these neurons were activated by increasing right atrial pressure. After phenylbiguanide injection, only 13% of the cells responded to the stimulation of baroreceptors and only 6% to cardiac mechanoreceptor stimulation. The author indicated that there was a high proportion of afferent convergence from pulmonary C-fibers, cardiac receptors, and peripheral chemoreceptors in the nucleus of the solitary tract. The role of pulmonary C-fiber receptors was considered to be inhibitory and a part of the defense mechanism, even though the exact role was still unknown.

The term expiratory flow limitation (EFL) is a physiological concept to indicate that maximal expiratory flow is achieved during tidal breathing (73). When we reach EFL during expiratory breathing, further effort to increase the expiratory volume cannot increase the expiratory flow rate due to intrathoracic airflow obstruction. The presence of EFL during tidal breathing promotes dynamic pulmonary hyperinflation (DH) and intrinsic positive end-expiratory pressure (PEEPi). This will cause an increase in respiratory work and an adverse change in hemodynamics commonly seen in patients with advanced chronic obstructive pulmonary disease (COPD). When EFL and DH develop, one can overcome EFL by increasing alveolar pressure and elastic recoil by increasing air through increasing inspiratory work. However, in excess, this may lead to people feeling like they “cannot breathe”. In patients with COPD with EFL, the DH will cause a further increase in inspiratory work due to air-trapping. In a study on the effect of bronchodilators on patients with COPD (74), the authors found that patients with COPD with EFL may experience less breathlessness after a bronchodilator during light exercise than those without EFL. This beneficial effect of bronchodilation occurs even in the absence of a significant improvement in forced expiratory volume at 1 s (FEV1). Grossly obese people appear to have EFL and PEEPi through small airway closure and air trapping (75). In the study subjects, EFL was more common when they were in a supine position, and tidal breathing was affected by EFL and PEEPi. A consequence of EFL will be an increase in end-expiratory lung volume, resulting in an increase in functional residual capacity (FRC) at the end of expiration. The PEEPi can impose an additional elastic load on the inspiratory muscles and thus increase the work of breathing.

The great deal of breathlessness an inexperienced swimmer undergoes is likely from an increased intrathoracic pressure and trapped air causing an increase in inspiratory work to generate the needed venous return, in addition to the activation of pulmonary C-fiber receptors. Talking in unstable postures necessitating torso stiffness with an increase in intra-abdominal pressure may bring a subsequent development of air-trapping and PEEPi. If this airway trapping and development of EFL and DH continues over an extended period of time, he/she may develop a functional impairment of inspiratory muscle and adverse effects on hemodynamics, resulting in shortness of breath (76).

## 6 Biomechanics of laughter and crying

Laughter, often said to be the best medicine, decreases tension (77) and anxiety and is not unique to humans. Laughter is instinctive and contagious, is a form of social play and vocalization, and is unusual in solitary settings. If loud laughter can be induced by tickling and a young infant laughs during a simple game of “peekaboo”, the primary role of laughter must be physiological rather than psychological. When rats were tickled to induce laughter, the laughing rats were more optimistic in making decisions when uncertainty was involved (78). Laughter among shelter dogs helped the shelter dogs to reduce stress and increase prosocial behavior, which could potentially lead to reducing residency time (79).

Developing delicate systems to avoid excessive and prolonged pressure on thoracoabdominal organs while maintaining necessary stiffness for postural maintenance would be a prerequisite to becoming successful obligatory bipedal primates. Young infants have to learn to control their trunks to sit and stand before they start walking and running with a much higher degree of trunk control (80). However, avoiding excessive pressure while learning trunk control will be a crucial step as we already discussed the harmful effect of sustained increased IAP. Although normal IAP of a child is reported as 7 ± 3 mmHg (81), a healthy newborn may even have a lower value. When sitting infants react to motions with a degree of trunk control, they may utilize a rapid decompression of air (laughter) that can lower the pressure inside the torso to protect internal organs and maintain bonding with parents since they are defenseless at this stage of life.

Laughter with its repetitive expiratory contractions (average frequency of 4.6 ± 1.1 Hz) causes a final drop in functional residual capacity (FRC) by 1.55 ± 0.40 L (82). As the diaphragm counteracts contracting abdominal muscles, this counteraction appears to protect intra-thoracic structures from the contracting abdominal muscles. Closure of the glottis at the beginning of each expiratory contraction slows down expiratory effort and increases intra-alveolar pressure. The contracting abdominal muscles increase both intra-gastric pressure (Pga) and intra-esophageal pressure (Pes), but the increase in Pga is much higher than Pes by an average of 27 ± 7 cmH_2_O during the entire fit of laughter. Contrary to trapping of air and dynamic hyperinflation during a stress response, a fit of laughter with the drop of FRC and the abdominal compression (with the pressure gradient between Pga and Pes) and following large negative inspiratory force will increase venous return and, in turn, cardiac output. In a study on the impact of laughter through humor on air trapping in severe COPD (83), the authors noted that laughter and smiling through humor intervention could induce a decrease in total lung capacity (TLC) by up to 1.55 L via reduction of RV (residual volume). FRC did not change. This indicated that there was a reduction in air trapping. Additionally, true laughter evoking more H-reflex suppression than simulated laughter (77) can be helpful in social interaction as the person is less guarded, more relaxed, and often falls off the chair.

When an individual encounters a stressful event or news and is unable to make a “fight-or-flight” reaction, crying seems to help avoid air-trapping and excessive intrathoracic pressure by contraction of abdominal muscles with narrowing of the laryngeal airway for enhanced sighing-like expiration. Subsequent inhalation after the contraction of abdominal muscles will induce an increased venous return to maintain circulatory volume. This contraction of the abdominal wall muscle during crying seems important for ventilation in a newborn with weak elastic recoil strength of the lung, In a study on expiratory muscle activity in preterm babies (84), the authors observed that the well-preterm babies used external oblique muscles during the expiratory cry but not during the intake of breath between cries. The grunting preterm babies were using external oblique muscles during grunting by forcing gas through the partially closed larynx which may help force gas into unexpanded regions of the lung. These expiratory abdominal muscle activities can help distribute force through the compliant lung areas to increase pressure inside the torso. The proper increase of pressure during crying, unlike laughing, can help maintain a degree of torso stiffness while avoiding dangerous air-trapping and hyperinflation and may be needed for posture control, unlike laughter. When a toddler falls after leaning on an unsecured object and starts to cry immediately, the toddler may get helped by extending his/her arms out by maintaining the hypertonicity of the torso (85). During temper tantrums in neurally underdeveloped toddlers who are unable to control ventilation upon stress, excessive outbursts of anger and crying may be compensatory strategies to handle ventilation and perfusion and would be worse if there are underlying deficits in inhibitory control, leading to future psychopathology (86). Although many illnesses cause pathological crying and laughing (87), crying and laughing are also interchangeable in many situations (i.e., award ceremonies). People accepting the award are likely to show their excitement and happiness through laughter first, but soon they will need to stiffen up in front of the audience while holding the trophy. The avoidance of excessive internal pressure can be achieved by laughing, crying, or both for a proper response to the given situation.

## 7 Predictive processing of perception

The reason that we cannot tickle ourselves seems to be the fact that the sensory consequence of self-generated movement is accurately predicted and suppressed. The amount of attenuation is proportional to the accuracy of the sensory prediction in self-generated movement with a narrow window (<100 ms) (88). Mechanisms to suppress or attenuate unnecessary information before it reaches the brain seem to be important to keep our attention on needed targets; otherwise, any such distractions would be dangerous for the survival of animals in the wild. Without proper prediction and suppression in vision, we will not be able to see at all during fast saccades due to the carried-over visual information formed on the retina (89). Indeed, we cannot see our own eyes moving in the mirror. Additionally, sensory inputs of unexpected events can be sensed acutely with a proper amount of attention, as toe cramps at night (which don't have an efferent copy from not being planned in the brain) are sensed as quite annoying.

Predictive processing is a well-accepted theory of perception and seems to be the main mechanism to enable us to engage in complex and fast social interaction (90, 91). Additionally, spontaneous activities (SA) of sensory areas of the brain in the absence of sensory stimuli appear to contribute to the perception and filtering of relevant inputs to integrate diverse sensory and non-sensory information to modulate behaviors and facilitate learning (92) with maturity (93). The complex neural sensory computation may be critical for the survival of animals, as any delay in perception and reaction by a fraction of a second can be deadly. Especially for large animals, including humans, on-site programming of action after computation of sensory input would not be compatible with survival in nature during hunting/escaping because there is a significant sensory-motor delay due to slow nerve conduction by distance and slow force generation of large muscle mass (94). For large animals, it can also be quite challenging to properly balance during fast locomotion if reactive jerks become frequent and cause perturbation of posture. For the mechanical property of large-bodied animals, predictive processing of incoming sensory signals of self and non-self origin would be a proper strategy for smooth and fast movement (95, 96).

Among many conditions, autism and psychosis are known to cause errors in prediction (97, 98) and anxiety-related symptoms in individuals (99, 100). Due to the presence of prediction error, people with deficits in higher level supraspinal computation would have a degree of torso stiffness from activation of TrA (44, 45) during various tasks, and might avoid those tasks in the future due to experience related to the negative impact on circulation and ventilation.

The theta waves in electroencephalography reflect the resting and default modes of brain activity (101) and appear during predictable motor activity (102). Easily predictable activities can be restful for the brain, like knowing the exact time of arrival of the last bus at a bus stop without a need for constant watch-out. Mind wandering (daydreaming) can also be restful for the brain from the fact that such activities do not require preparation for the pre-programmed motor activity of abdominopelvic muscles (24, 26) and arrangement of real-life predictions and monitoring of outcomes, thus allowing for more theta activity of the brain (103). Relaxation during watching clouds or far scenery would be from the fact that there is no need for immediate motor planning and monitoring. Maladaptive daydreaming is known to be linked to various psychopathological symptoms (104) and may reflect the underlying struggle to ease the tension of the torso for circulation and ventilation. Various habits (including nail-biting and lip-biting) would be easy real-life motor tasks, as motor actions on oneself are very predictable. Simple motor activity in a state of stable balance (like knitting while sitting) may induce relaxation of the diaphragm since it no longer needs to take on a postural role in humans (24) and promote theta waves from increased predictability of ongoing action. Many rituals can be helpful to ease one's tension due to their predictability (if practiced and familiarized) and used more during stressful times (105). In the same sense, Western classical music of highly regular and predictable rhythms may give us relaxation (106), and the predictability of the next note in the music being listened to increases as it is played repeatedly. The reverberating resonance in low-pitch meditation music may do the same, as the following sound becomes predictable due to the lasting resonance.

## 8 Vision and inhibition of return

Visual information is important for learning, prediction, and guidance of body actions in everyday activity (107, 108) with temporal guidance in space (109, 110). While the status of external ocular muscles appears to influence vestibulo-ocular reflex (111, 112), which is known to be important for balance, significantly, gaze direction seems to guide cervical muscles (113) and override vestibular signals for postural motor responses (114). Oculomotor deficit, which is common in many psychiatric illnesses including anxiety disorder (115) and autism (116, 117), may affect learning and executing motor activities negatively in everyday life.

While viewing behavior is quite biased toward given tasks (118), the clear foveal vision is only 1–2 degrees of the visual field with rapidly fading retinal images, and the need for redevelopment of perfectly-fused binocular retinal images with frequent saccades seems quite challenging when there is constant often irregular movements of self and others. Proper allocation of visual attention (119) with an accurate prediction of the future location of moving targets (120) can be important for complex social activity and is critical for animals to search for food and avoid predators.

Inhibition of return (IOR) is a well-known concept in vision research and states that response time to previously attended areas is slower. This inhibitory mechanism allows a person to allocate visual attention to unattended areas (121). Having a degree of deficit in IOR can have a negative impact on fast learning in complex social activities. Due to errors in prediction (97) with deficits in IOR, people with autism may have significant torso tightness (44, 45) from lowered predictability and perceptions of unexpectedness (46) in challenging environments, and may feel worse during actions including gait and language which would get influenced by less efficient gaze patterns for the guidance of motor activities (107–110) and postural balance (112, 113, 122). Deficits in IOR in obsessive-compulsive disorders (123) and attention deficit hyperactivity disorders (124) can be presented as anxiety during social activity if the allocation of visual attention is impaired in a complex and rapidly changing world.

## 9 Common conditions related to anxiety

### 9.1 Vasovagal syncope: neurally mediated syncope

There are many reasons for syncopal attacks, but a neurally mediated type is the most common and quite prevalent in the general population. Although the exact pathophysiological mechanisms of neurally mediated syncope (NMS) are not well known, the Valsalva maneuver can induce lightheadedness by blowing hard against the closed vocal cords to generate positive intrathoracic pressure. During the resisted expiration procedure to bear down the force meter at 20 cmH_2_O for 7 s, both end-diastolic and end-systolic volumes of the left ventricle fell precipitously during the strain phase of the procedure with a concomitant decline in cardiac output (53). These findings suggested that the change in cardiac performance was due to the altering of ventricular filling and changing afterload. However, mildly-resisted inspiration, unlike resisted expiration, improves cardiovascular performance and tissue perfusion through a decrease in intrathoracic pressure and an increase in venous return (125–127).

During the tilt-table test (used to diagnose NMS), a patient in a supine position with a foot plate is secured by straps and belts. After the patient is fully upright, the table is then gradually tilted backward at a 60–80 degree angle. Sometimes, vasodilator agents are infused intravenously to increase the sensitivity and specificity of the test. A positive (abnormal) test result is characterized by a loss of consciousness that follows various hemodynamic patterns including hypotension with or without bradycardia. Even though the test is considered a safe procedure and an asystolic pause itself is considered a positive response, extremely prolonged asystole has been reported (128, 129).

While skeletal muscles pump venous return (60) via the eccentric contraction of large posterior posture muscles during the weight-bearing stance phase of gait (130), a restrained person in a tilted backward position after the initial upright standing will not be able to recruit large eccentric posterior postural muscles. This tilted posture mimics the non-weight-bearing swing phase of the legs to limit venous return. Further, straps and belts applied to the front torso will limit outward inspiratory movement which is important in restoring circulatory volume in hypovolemia (126) and hypotension (125). If a susceptible person is on the table for a prolonged period and activates torso muscles (including the diaphragm, TrA, and pelvic floor muscles) for the needed stiffness of the spinal column (24, 25, 27, 28, 131), this will result in a further deterioration of venous return.

In a Mueller maneuver (opposite of the Valsalva maneuver), the increase in abdominal pressure using rib cage muscles can briefly increase the venous return from the abdomen (132). This mechanism can be seen during “sighing breathing”. However, a prolonged increase in IAP will cause a drop in venous return which, in turn, will decrease the volume of the left ventricle. The static contraction of the diaphragm during continued thoracic breathing for postural control will inhibit the elastic recoil of the lungs upon exhalation and cause air-trapping with dynamic hyperinflation. At higher lung volumes, the expiratory effort will cause a much higher intrathoracic pressure due to air-trapping and dynamic hyperinflation (132). Because of the higher lung volumes, the greater expulsive effort needed for expiration may induce the Valsalva effect (like blowing a balloon). When thoracic breathing (inspiration and expiration) at higher lung volumes causes a decrease in venous return and an increase in intrathoracic pressure, there will be a compressive effect on pulmonary capillaries (4–12 mmHg), right atrium (2–6 mmHg), and left atrium (<13 mmHg) with a subsequent decrease in left ventricular volume (dimension) and cardiac stroke volume (133). Infusion of vasodilating agents (including many antihypertensive drugs) will cause a decrease in preload and afterload to contribute to the pathogenesis of vasovagal syncope.

A necessary increase in adrenergic tone, when preload is low, can induce poor relaxation of muscles and further limit cardiac filling. If excessive adrenergic flow into the cardiovascular system by the activation of afferent baroreceptors is not counterbalanced, the excessive cardiac contraction with decreased venous return (underfilling) could cause an over-shortening of the sarcomere of left ventricular muscles at the end of systole (the beginning of diastole). This overshortening of sarcomere with decreased diastolic volume could allow “contraction band” formation (hypercontracted sarcomeres) which can be seen in “Takotsubo” cardiomyopathy (134, 135) and in the cardiac death of stranded animals (55, 136).

If there is an activation of pulmonary C-fiber receptors as a result of increased intrathoracic pressure (which causes bradycardia, hypotension, and a decrease in muscle tone), then the loss of consciousness (syncope) can be seen as a defense mechanism rather than an abnormal neuro-humoral reaction (like a circuit breaker in a system overload) as spontaneous breathing is beneficial over positive-pressure ventilation during hypotension (126) if maintained during syncope.

The vasovagal reaction seems to happen even after heart transplantation (137) because of the existing defensive role of pulmonary C-fiber receptors rather than paradoxical stimulation of left ventricular baroreceptors from neurohumoral dysfunction (or the reaction should not happen after denervated transplanted heart). In this study (137), patients after heart transplantation underwent head-up tilt (up to 60 degrees) testing while resting on a saddle support (bicycle saddle fixed on a steel tube which prevents proper weight-bearing for venous return and posture). During the tilt with saddle support, the patients could induce the activation of torso muscles to increase IAP and induce air-trapping with dynamic hyperinflation.

Selective Serotonin Reuptake Inhibitors (SSRIs) appear to prevent vasovagal syncope in patients with refractory NMS (138). Although negative conversion of the tilt test seems to support the efficacy of the drug on the neural mechanism, people treated with SSRIs still continue to have a positive response to lower body negative pressure (inducing decreased venous return by venous pooling) during the tilt test (139). These findings appear to indicate the possible role of SSRIs on NMS through the change of motor (behavioral) patterns during stressful situations rather than through the modification of the sympathetic tone or its withdrawal as serotonin is known to modulate the central pattern generators (CPGs) in animals (140–142).

### 9.2 Takotsubo cardiomyopathy (stress cardiomyopathy: broken heart syndrome)

Although many people assume that the heart pumps blood out by shortening the length of the sarcomere (concentric contraction) with widening muscle fibers based on sliding theory, if this were the case, then the concentrically contracting cardiac muscles yield 11.6% work with the rest lost as heat energy (143). While moderate heat stress is already detrimental to cardiac conduction (144), if the heart beats >100,000 times a day with that much heat, survival is impossible. For this reason, there must be other mechanisms to prevent excessive energy use leading to fatigue and rigor. Unloaded shortening-induced deactivation by the structural change of the physical state of the regulatory protein complex of the actin and myosin units, not due to the depletion of chemical energy, seems important for energy saving in skeletal muscle physiology (145). In cardiac muscles, the recoil moment of stretched elastic titins upon unloading seems to contribute to the mechanical deactivation of calcium-dependent acto-myosin bindings to spare ATPs (146). Obliquely running elastic regions of cardiac titins can provide a longitudinal and radial force that compresses the lattice upon lengthening (147). After the initial shortening of fibers upon the start of systole, through the unique spatial arrangement (148) and radial branching pattern of cardiac muscle fibers, there is a lengthening event during cardiac contraction with geometric alteration in fiber and sheet structures to induce circumferential shortening and regional wall thickening rather than an increase in fiber size (149, 150). However, when preload is low with an adrenergic surge during stressful situations, a degree of concentric contraction forming contraction band may occur (54). This may explain the sensation of internal “heat” in many people with recurrent syncope just before they pass out.

Takotsubo cardiomyopathy was once thought to be rare but now seems to have been an under-recognized condition with increasing numbers of case reports in various countries (151). In a tertiary hospital, it may account for approximately 2% of hospital admissions for acute coronary syndrome (152). Even though a complete recovery is expected in the majority of cases, it can be fatal. It is an acute illness with a sudden onset of chest pain and shortness of breath, usually triggered by an emotionally or physically stressful situation. It appears to happen more often in women over 50 years old. When they present to an emergency room for chest pain and dyspnea, the initial findings are suggestive of acute coronary syndrome with changes in EKG (electrocardiogram) and blood biochemical testing including elevation of cardiac enzymes. However, objective findings usually fail to show any significant coronary atherosclerotic stenosis, coronary vasospasm, or myocarditis (134). Instead, the patients with this condition show a distinctive pattern of apical akinesis of the left ventricle in angiographic images which is similar to the shape of a fishing pot used for trapping octopus in Japan.

If a sudden severe (emotionally or physically) stressful situation causes a severe adrenergic response, the required postural stiffness will increase intraabdominal and intrathoracic pressure. Suppose this reactive stiffness is not compensated by physical movement (fight-or-flight) and proper ventilation. In this case, there will be a significant drop in intraventricular volume (53) from decreased venous return and extrinsic compression on the cardiac chambers and vena cava veins (153). If pulmonary C-fiber receptors are not activated immediately or are overridden by supraspinal (cerebral) command (70), excessive and continuous contractions of the left ventricle under the adrenergic influence with decreased preloads will cause over-shortening of muscular fiber length to form “contraction bands” from hypercontraction of sarcomeres (134, 135). This hypercontraction of cardiac muscle fibers may be the cause of the chest pain (cramps) and happens more at the apex sparing the peri-valvular area due to its valvular attachment.

If unopposed to the excessive adrenergic surge with hypercontractility, the unsecured freely moving apical cardiomyocytes can develop rigor and contraction bands unlike the secured basal cardiomyocytes. The unique inhibitory mechanism of apical cardiodepression for cardiac protection at the time of adrenergic surge seems necessary (154) to avoid myocardial toxicity and contraction band formation and may give the unique shape in the ventriculogram by the time an angiogram is done. The extremely high level of catecholamines (much higher than in normal stress response) can be the result of cardiac pump failure (135); however, the perfusion pressure to the vital organs may be maintained by catecholamines increasing the contractillity of basal cardiomyocytes (154).

### 9.3 Postural orthostatic tachycardia syndrome

Postural orthostatic tachycardia syndrome (POTS) is considered an autonomic dysfunction that causes postural lightheadedness, fatigue, sweating, tremor, anxiety, palpitation, and near syncope (155). People with this condition have an increased heart rate > 120/min (or increase by 30 from resting heart rate) after standing for 10 min. The condition is more common in women (5:1 female to male ratio) in the age range of 12–50.

If a person develops patterns of excessive activation of torso muscles with an increase in intra-abdominal and intra-thoracic pressure, if the patterns are sustained with an increase in adrenergic tone but limit splanchnic reserve of blood volume (156), and if the patterns are maintained into daily life activities with a suppression of pulmonary C-fiber activation by supraspinal (cerebral) override (70), the patterns causing decreased venous return and insufficient intrathoracic blood volume (153) will not be sustainable. With a limited splanchnic reserve and an impeded venous return, an increase of sympathetic stimulation of the heart alone will result in only small increases in cardiac output (156). If activation of baroreceptors with hyperadrenergic vasoconstriction is prolonged (153), irreversible myocardial injury with ATP depletion can occur (54).

However, the sympathetic outflow and vascular resistance of the skin are not regulated by baroreceptor activity (157) but rather by central motor command (158). The activation of cutaneous sympathetic tone promoting sweating and flushing may help to prevent hyperthermia during hyper-adrenergic situations. While sitting down alleviates POTS by preventing excessive torso stiffness and bringing the venous pool closer to the torso, enhanced inspirational drive via common respiratory symptoms (159) when preload is low (160, 161) can expand central blood volume to ameliorate orthostatic hypotension (125, 162). POTS seems reactive to the circulatory compromise by an extended adrenergic response during improper upright activity rather than an autonomic dysfunction.

### 9.4 Abdominal pain and irritable bowel syndrome

These are common symptoms related to anxiety and often become severe enough to make an emergency room visit. Contrary to the severe pain causing “doubling-over” posture and tenderness requiring intravenous pain medications, laboratory and imaging tests often fail to reveal any significant pathologic findings to explain the severe pain (163). Other people with discomfort in the pelvic or suprapubic (bladder) area with urinary frequency or urgency and sometimes painful urination are often treated for urinary tract infections (164). It is also common for many people to have bacteria in their urine without any symptoms. Occasionally, a normal urine test is obtained when the clinical presentation is identical to prior urinary track infection events. Referral to urology is often made and patients undergo a series of tests with a diagnosis of interstitial cystitis. Sometimes, surgical implantation of a sacral nerve root stimulator is attempted with varying results (165).

A motor unit consisting of a motor neuron and all the muscle fibers it innervates can have a varying number of muscle fibers per motor unit (from a few to several hundred) and generates force determined by the number of muscle fibers in the unit. During muscular activation, because all muscles consist of many individual motor units mixed amongst fibers of other units, activation of one motor neuron can result in a weak but distributed muscle contraction. There will then be subsequent activation of more muscle fibers when additional forces are needed, known as motor unit recruitment (166). Because the motor unit recruitment reflects how many motor neurons are activated in a particular muscle, it can be used as a measure of the muscle contractile force. The larger the recruitment, the stronger the contractile force will be. The contractile force can also be increased by the motor unit firing rate. The motor unit firing rate of each individual motor unit can increase with increasing muscular effort until a maximum rate is reached.

In slow and low-amplitude muscle contractions, a minimal firing rate modulation of the early recruited (low threshold) motor units appeared to be due to an inhibitory mechanism by the newly recruited motor units (167). The motor units recruited later showed depression of active firing rates when additional motor units were recruited for higher force development. In linearly changing voluntary contractions (168), recruitment is the major mechanism at low levels of force, but increased firing rate becomes the more important mechanism at intermediate force levels. A brief voluntary contraction superimposed on sustained contraction can induce a short suppression of low-threshold motor unit activities. This recruitment, de-recruitment, and re-recruitment appear to represent a mechanism to reduce fatigue of motor units during sustained contractions (169). However, during static contractions and slow (and low degree) dynamic contractions of low force (10% of maximal voluntary contraction force: common in occupational activities), some early-recruited motor units had continuous activities without rotating bursting and depression seen in brief voluntary contraction (170). This indicates that there are many motor units prone to fatigue and metabolic injury during prolonged repetitive work with insufficient pauses for a full recovery.

During the contraction of muscles causing tissue constriction, it is surprising that resting intramuscular pressure is below 5–10 cmH_2_O and intramuscular pressure can rise rarely above 20 cmH_2_O during voluntary contraction (171). However, maintaining proper blood flow into the feed arteries, intramuscular arteriolar networks, and capillaries with proper coordination of the vasoconstriction and vasodilation will be important to avoid tissue ischemia resulting in injuries. In a study on vasomotor tone in resistance vessels of hamster pouch retractor muscles, the authors found that there was a progressive constriction of arterioles and feed arteries with muscle lengthening (within a physiologic range) which decreased blood flow >50% (172). The reciprocal relationship between muscle length and the diameter of arterioles and feed arteries was sustained across muscle lengths. These length-induced vasomotor responses were triggered by norepinephrine release from perivascular sympathetic nerves within the retractor muscle, independent of the central nervous system (CNS). They concluded that intramuscular and extraparenchymal resistance vessels actively respond to mechanical forces within the muscle, independent of muscle fiber activation or the release of vasoactive metabolites. While the passive extension of the retractor muscle activates periarteriolar sympathetic nerves, muscle contraction (with acetylcholine release by the motor nerve terminals within the muscle) evokes (ascending) arteriolar dilation in a reciprocal manner, which is then conducted back into feed arteries (173). With feed arteries located next to skeletal muscles giving rise to intramuscular arteriolar networks, the mechanotransduction sequence via the change of length of skeletal muscle is integrated by the neural regulation of feed artery (and arteriolar) resistance and the supply of oxygen to muscle fibers (174). The changes in the cardiovascular system (which is mediated by hormones, reflexes, and CNS drive) also appear to be graded according to the degree of muscular activity and the volume of muscle involved (175). Venules, like arterioles, dilate actively in response to muscle contraction (176). This dilatation will help reduce the rise in capillary hydrostatic pressure to limit the outward filtration of fluid.

Because local mechanisms via length change are the most important mechanism for muscle perfusion (172–174, 176), prolonged static sitting with the silent psoas muscles (38) will recruit the abdominal wall muscles for the stabilization of spine (27) and resulting ischemic injury to the involved motor units would be inevitable. Biopsy specimens of work-related chronic myalgia showed pathologic “ragged red” fibers which are usually found in ischemic injury to the mitochondria (177), with decreased levels of ATP and ADP indicating reduced muscle oxygenation (178). Tissue doppler of the area of pain showed an impaired local capillary blood flow in the tender part (179). If a similar ischemic change is to happen in abdominopelvic muscles over a prolonged period of time, painful afferents from motor units of local ischemic injury [especially the transverse abdominis (TrA) muscles with diffuse attachment and no directional vector unlike other limb muscles with joint motion] may not be suppressed to give an impression of intraperitoneal pain. The severe pain perception can be a mismatch of expectations by the lack of cerebral efferent of the cramping action (like toe-cramps while sleeping) and related stress response to the unexpected afferents would incur further tightening of the postural muscles involved in vicious cycles. It is known that the cramp threshold gets lower in subsequent cramping contractions (180) to make the situation much worse.

During prolonged stress posturing while sitting under demanding tasks, additional ischemic injury to highly aerobic intraperitoneal organs is expected due to sustained elevation of IAP. Abdominal bloating, dyspepsia, and diarrhea are common symptoms related to irritable bowel syndrome. If the diaphragm, TrA, and PFM are to contract for stress response to raise IAP for posture control, the compensatory relaxation of internal oblique muscle and upper rectus muscle will be necessary (181) which can be seen in CT scan images of patients with functional abdominal distension as the descent of diaphragm without change of intra-abdominal volume (182). Then, bloating with diarrhea can indicate a degree of ischemic strain to internal organs during stress posturing and also serve as a compensatory mechanism to decompress the peritoneal cavity from the strain. Compared to the thoracic inspiration which is common in stress response, the cyclic diaphragmatic inspiration with a higher pressure gradient by its descent into the intraabdominal cavity (30, 183) can be an efficient way to drain venous blood and lymphatic fluid and then allow the passage of the incoming perfusion to the internal organs. The static abdominopelvic muscle strain with prolonged thoracic breathing causing ischemic injuries has to be considered in various organic inflammatory disorders, as stress is known to be the most important trigger in ulcerative colitis and Crohn's disease (184).

Central pattern generators (CPGs), producing rhythmic outputs in the absence of rhythmic input, seem to be the source of rhythmic and stereotypical behaviors, like walking, scratching, chewing, and breathing. Through the contributions from CPGs, we humans can make rapid adaptations to environmental changes and store new patterns very quickly (185, 186). Likewise, the ability to recruit different patterns quickly upon unexpected outcomes seems critical to avoid falls (187) and injuries (188). Patterned startle responses, such as in a common scenario when one might make a misstep going up or down a flight of stairs, appear to be context-specific (189) and site-specific (190). Besides central sensitization of pain hypersensitivity (191), if patterned stress posturing recruits motor units with ischemic injuries upon recurrent exposures to similar stressful situations, the motor afferents from ischemic motor units will be sensed as “unexpected” to cause more painful contractions.

A similar etiology may apply to chronic pain related to fibromyalgia which is also associated with an increased risk of irritable bowel syndrome. People with a history of chronic pain of ischemic/myalgic origin will have a significantly lower cramp threshold, which can be even lower after painful cramping contraction of the affected motor units (180). During a painful contraction (sensed as an “unexpected” afferent), the “unexpected” pain sensation will not be suppressed unlike how we suppress our expected senses during everyday activities (88, 192). Although many consider the pain in fibromyalgia to be neuropathic in origin, the proper maintenance of the nerve endings embedded in the muscles will also depend on local perfusion to the muscles, mainly by length changes. The structural change of the brain is common in patients with chronic pain syndrome (but not specific) and is thought to be secondary to frequent pain stimuli. It may also be reversible when the pain is well controlled (193). The preservation of the corresponding brain cortex of chronic pain indicates that pain generation is a bottom-up process rather than a top-down process (194, 195). Due to its nature as a bottom-up process, chronic pain can be perceived as severe from lacking cerebral efferent copies.

## 10 Discussion

To understand the pathophysiology of anxiety, the importance of venous return in circulation needs to be considered because the heart can pump only what it receives. While weight-bearing activity is the most important mechanism for venous return (60), proper breathing (61) through careful control of the torso is needed during upright bipedal activities. Successful bipedal locomotion with an inherently unstable spine (47, 48) and significant sensory-motor delay for force generation (94) must depend on accurate prediction and acquisition of many patterns (pre-programming) while simultaneously avoiding unnecessary torso stiffness. This must have been a prerequisite in human evolution. Through CPGs and preprogramming, we can perform fast motor activities without cognitive delay even before the active top-down supraspinal command (196). Indeed, we feel clumsy and slow to learn new skills using new tools in new environments.

It is interesting that, in addition to their failed adaptation to cold climates (197), the extinction of the Neanderthals might also be attributable to preeclampsia (198, 199). During pregnancy, torso control will be even more challenging due to the rapid growth of the large fetus and significant proprioceptive challenge from the loosening process of joints and muscles. If not controlled carefully, excessive pressure in the torso can impede venous return and circulating intravascular volume. Rapid fetal growth and exponential growth of the cerebellum in the large fetal heads in late pregnancy need a secure blood supply through the invasive placenta to the mother's womb. The disturbance of its growth in premature infants can cause subsequent neurocognitive and behavioral deficits (200) including autism (201). The vascular hallmarks of preeclampsia are placental arteriolar narrowing and fibrinoid necrosis which are likely reflecting the mother's struggle to preserve her circulatory reserve for her own survival although the invasive human placenta on the uterus is supposed to secure the necessary blood supply for the developing fetal brain.

Although the large pelvic opening of modern humans with pelvic dimorphism allows the passage of the fetal head, the large fetal head is still problematic and requires rotation of the fetal head during descent in the birth canal, unlike other primates (198). The large size of the Neanderthal's fetal head might have been problematic, similar to modern humans. Unlike modern-day humans, the Neanderthals had bigger pubic bones and wider pelvises without sexual dimorphism (202) with shorter lower limbs (203). This might have allowed them to be adept at activities of the upper body in their environments near glacial perimeters (204, 205) and wooded sloped terrains (206). They might have engaged in many anaerobic activities using the upper body, unlike our ancestors from the African savanna. Both the Neanderthals and modern humans were able to use sophisticated weapons technology and well-coordinated group hunting skills with the knowledge of the anticipatory behaviors of prey animals (207). However, it appears that modern humans were able to exploit much larger areas, establish broader social networks, and use local and non-local materials compared to their Neanderthal counterparts (207). This locomotive advantage with broader social networks can be supported by the energy-efficient modern human locomotion (22), unlike the Neanderthals of high daily energy demand (208) with wider pelvises and stocky bodies. Hyperadrenergic stress responses (209) with limited aerobic muscle activities in cold, wooded, and sloped environments (204–206) might have been deleterious to the societal bonding of the groups and the fetal development in pregnant females, as we know that stress can impair the prefrontal cortex structurally and functionally (210) and fetal development (211, 212). Further, the paternal experience of stress seems to have a lasting effect on future generations through transgenerational epigenetic inheritance (213).

Women of childbearing age with wider pelvis orifices and an open-plan arrangement of pelvic floor muscles (compared to their male counterparts) would require an increased tone of abdominopelvic muscles to support intraperitoneal organs during upright activities. Increased adrenergic tone related to an increased torso tone would cause a degree of venous impedance in women of childbearing age. Progesterone is known to have an inhibitory effect on muscle contraction (214, 215) and peak saccadic velocity is diminished during the luteal phase (216). The acute withdrawal of progesterone in the premenstrual period may have effects on proprioception and gaze control resulting in errors in motor planning and sensory monitoring (217). This would be much worse in susceptible individuals with underlying oculomotor dysfunctions and prediction errors (218) because gaze has an important role in guiding everyday activities (108) and postural stability (114), reflects decisional preference (219), and can be manipulated to bias one's decisions (220). Various circulatory symptoms may occur if prediction and monitoring functions are altered because gaze, sensory discrimination, and action execution are coupled to cardiac cycles (221–223). Individuals with convergence insufficiency which is a common oculomotor dysfunction affecting 5% of the population with limited reflective convergence capacity at near-point viewing (224) would need to over-fixate eyes on near-point screen tasks to cause strain and fatigue on delicate ocular muscles, likely from the overuse of the superior rectus muscles which are recruited for needed convergence and connected to the adrenergic system. Prolonged strain and ischemic injury may cause errors in saccades accuracy for visual learning and guidance which may contribute to many psychological symptoms (225) from unfavorable patterns of viewing of a complex world.

The level of proinflammatory cytokine IL-6 increases during stress responses including social and psychological stresses (226, 227). Increased IL-6 level is found in many psychiatric conditions (227–230). The skeletal muscles become a major source of IL-6 (231), and its production can increase by muscle damage (232), excessive and fatiguing exercise (233), low pre-exercise glycogen content from prolonged exercise with limited rest (234), and insufficient nutritional intake (235). Endurance training resulting in improved performance (236) and glucose ingestion (237) attenuate IL-6 release from contracting skeletal muscles. However, forced exercise does the opposite (238). During aerobic exercise, the hepatosplanchnic viscera appears to remove IL-6 from circulation to limit the negative effect (239). Elevated IL-6 level is also found in chronic myalgia (240) which is known to have local hypoperfusion and ischemic changes (177–179) as in other chronic illnesses. Particularly, local ischemia seems an important trigger of IL-6 production (241, 242).

While IL-6 promotes the production of other cytokines related to atopy and asthma (243) and causes myocardial failure and skeletal muscle atrophy dose-dependently (244), it also crosses the brain-blood-barrier and placenta. In animal models, a maternal injection of IL-6 mediated the socio-behavioral deficit (such as autistic behavior) in offspring, but co-administration of anti-IL-6 antibodies prevented the deficit (245). Although the cause of autism is not well known, elevated levels of maternal IL-6 linked to prenatal maternal stress may contribute to the risk of autism in humans (246, 247). The increased adrenergic tone from stress during pregnancy affecting circulating volume may cause ischemic strain and elevated levels of IL-6, which in turn may contribute to an increased risk of preeclampsia and autism (201). Likewise, prolonged static sitting with poor venous return during demanding tasks may affect systemic perfusion in the same way in pregnant women. Autism is frequently related to hypoplasia of the cerebellum, which is critical to coordinate ongoing motor actions with a precise prediction of the immediate future events of the self and surrounding environment (248). Being able to precisely put one's attention to the necessary location in response to cues and quick error correction upon mistakes is critical for the development of social skills. A delay or deficit of these skills will prevent the development of social skills from fragmented information in fast-changing environments (248) causing social anxiety and avoidance of social interactions.

Aerobic activities improving tissue perfusion through increased cardiac output (cardiac index) and decreased peripheral vascular resistance are beneficial to many conditions associated with anxiety and panic disorders (10, 249–252). Aerobically working muscles seem to work in tandem with the diaphragm to be the major pump for circulation over the heart when we consider the following: First, the stimuli to the cardiovascular responses to exercise come more from the muscles themselves than others (hormones, reflexes, and CNS drive) (175). Second, proper coupling of vasodilation and vasoconstriction within near the active muscles through cardiovascular adaptation (change in blood pressure and heart rate) are graded according to the degree of muscular activity and the volume of muscle involved (175). Third, muscle perfusion depends on local mechanisms mainly through muscle length change (172–174). Lastly, venules (like arterioles) dilate actively in response to muscle contraction to reduce the rise in capillary hydrostatic pressure to limit the outward filtration of fluid (176).

A typical municipality in the U.S. spends about 25–40 percent of its total energy bill on drinking water and wastewater systems to provide safe drinking water, and 90%–99% of energy consumption at a water system either using groundwater or surface water is primarily due to pumping (253). A water tower generating sufficient pressure to deliver water relies on proper pumping with pressure monitoring, and prolonged pumping failure in a municipal water system would make the town uninhabitable.

The “lactate shuttle” is now a well-accepted concept that explains the significant roles of lactate as a major energy source, a major gluconeogenic precursor, and a signaling molecule with autocrine, paracrine, and endocrine-like effects (254, 255). A large amount of lactate in the circulation is produced by the skeletal muscles during aerobic activities (254–257) and is an important and preferred fuel for the human brain (258–260) and heart (261). Lactate also reduces inflammation and organ injury (262, 263), and has free radical scavenging and antioxidant effects (264). Since humans have relatively large aerobic muscle mass compared to other primates (265), proper perfusion to those muscles would be necessary by frequent activity (266). However, modern-day lifestyles with excessive sitting seem to do the opposite (267, 268), resulting in a lack of the main fuel and antioxidants for the brain, the heart, and other organs in addition to spinal instability (38, 39) and activation of the abdominal wall muscles (44, 45). Further, if predictive processing is the main mechanism to perceive the world, proper brain computing is necessary for accurate perception of the world, and the accurate perception is essential for the prediction of higher probability and lower error rates; the benefit of sufficient aerobic activity to maintain and improve brain function can go far beyond cardiovascular benefits (10, 249, 269–278).

Further, our locomotive behavior on the pavement with a head-up posture (nice-and-tall) seems unsafe in natural environments with many obstacles if we walk barefoot. Proper visual attention (107–110, 279) is needed for balancing (113, 114) over many ground obstacles via accurate perception and prediction (like hammering a nail). The head-down flexion posture for visual guidance will activate posterior spinal muscles before the flexion-relaxation phenomenon (40, 280) to help the unstable spine (47, 48) by tensile eccentric contraction (281) to improve stability and balance (282, 283), analogous to cable grips or the counterweight systems of elevators and ski lifts. This can help free up the front abdominal muscles (40) for easier ventilation. Although alternating leg movement in human gait seems like passive motion, the swing phase is achieved by complex motor control to perfect dynamic synchronization and to utilize elastic restoring torques (284) which must integrate accurate prediction of passive tension. One reason for the benefits of barefoot walking over shod walking (285–288) on uneven natural ground can be from leaning forward to locate visual targets in time. Subsequent measured lifting and controlled landing of the swing leg mass occur for proper weight loading and dynamic synchronization. This differs from flat, paved surfaces with limited visual guidance where the psoas muscles get immediately inactivated after the initial swing phase (38) instead of stabilizing the spine (39). Enhanced balance via barefoot walking (285–287) may positively affect the activation of postural muscles (289, 290) to lessen anxiety-related symptoms (291, 292) as anxiety is linked to a deficit in balance (293, 294) and poor balance suppresses cardiac function and activates sympathetic tone significantly (295, 296).

Improper weight bearing on pavement can affect the venous return and circulating volume negatively and may contribute to anxiety in our society that is plagued by prolonged sitting and excessive near-point activities. On the other hand, playful aerobic activities in natural environments would make it hard to use existing motor patterns habitually built on pavements and might promote sensory integration for better motor outcomes (297) to improve anxiety (298–301). A similar principle may apply to the benefit of animal-assisted therapy (302, 303). If exposure to the natural environment and playful aerobic activity cannot be applied enough, obsessive thinking as a maladaptive daydreaming and compulsive behavior as a predictable activity may help ease tension and torso stiffness for the moment.

Severe emotional experience during a panic attack can pose a serious risk of cardiovascular events in susceptible individuals (coronary heart disease, Takotsubo cardiomyopathy, or sudden cardiac arrest) (3, 304–306) in young and old (307, 308) with uncontrolled anxiety disorder. Self-harming behaviors often associated with OCD (4, 5, 309), if not suicidal, can be seen as desperate efforts to restore circulation through a highly attentive, precise, and predictable action on oneself at the moment with subsequent physical withdrawal from the painful outcome. This may be more common in people with underlying prediction and coordination errors (310, 311). If our brain is optimized for the perception of the immediate future through predictive processing and spontaneous activity (90–92), frequent panic events caused by ongoing anxiety might influence the brain to predict and prepare for one's death which could be interpreted as suicidal ideation and attempts (6, 7, 312, 313) which are not explained by depression (6, 7). Suicidal ideation and an attempt would be more common in people with underlying prediction errors (314, 315). It is possible that suicidal ideation and even planning may function as maladaptive daydreaming to ease ventilation and perfusion if panic symptoms are not well controlled. Low dose opioid and vagal nerve stimulation are known to bring a prolonged expiration and an increased tidal volume (316, 317). Increased venous return from the splanchnic and non-splanchnic vascular beds (31) and improved cardiovascular function (29) are expected by enhanced respiratory pump (125, 126) with lowered intrathoracic pressure and may contribute to lowered suicidality (318–320). Considering the hypofunctioning prefrontal cortex during hyperadrenergic crisis (210), more prompt approaches may be necessary in the treatment of severe panic disorders over the step-wise approach to prevent irreversible outcomes from poor cognitive judgements.

## 11 Conclusion

The brain cannot function well if the heart pauses, and the heart cannot function well if venous return pauses. A proper amount of aerobic activity (60) coupled with quiet breathing (61, 321) is important for venous return and circulation to organs including the brain and the heart. The physical manifestations of feeling anxious are related to circulatory compromise and muscular stiffness which will also impede circulation by affecting skeletal muscle pump and respiratory muscle pump negatively. The reason that various methods including physical activity (12) and quiet breathing (321, 322) ease anxiety-related symptoms seems to be by enhancement of central circulation.

However, the contribution of abdominal muscles as an auxiliary heart (32, 33) can be significantly constrained if proper control of torso muscles is limited by various causes, intrinsically, extrinsically, or both. Considering the unique roles of the human diaphragm in posture control and ventilation, accurate prediction of sensory-motor outcomes and proper allocation of attention seem essential for the complex obligate bipedal activity. The predictive role of the brain in perception will be critical to overcome the significant sensory-motor delay. Through the complex learning process and pattern development, we humans can perform various motor activities (from walking to complex social and sports activities) efficiently with proper allocation of attention. Any delay in reaction or improper allocation of attention can be detrimental. As many conditions with prediction error present as spectrum disorders, various graded therapeutic activities can be considered to treat anxiety.

Playful aerobic activity for the skeletal muscle pump and proper ventilation for the respiratory muscle pump with a biomechanical approach and behavioral modification need to be considered as the first line of treatment and prevention of anxiety rather than adjunctive therapy (12). Our society has reduced playful aerobic activity dramatically with an increasing emphasis on academic competition and accomplishment, which inherently involves excessive static near-point activity and screen time. Playful aerobic exercise can also provide an important fuel and antioxidant to the brain via the “lactate shuttle” mechanism. Although our society promotes competition (which incurs stiff emotions unlike caring and giving) and exceptionality, exceptionality is often linked to deficits in social skills with a possibility of resultant overcompensation in the areas where functional individuals can make better predictions; some of these individuals might be labeled as “Gifted” (323–325). Promotion of yielding over competition seems needed to limit many harms from excessive anxiety as we feel comfortable when the outcome is predictable with less competition, like yielding over competing for a lane change while driving. Rapid increases in anxiety among young adults (326) and exponential rise in the recorded cases of autism (327) which is characterized by prediction error and anxiety may indicate that our society is failing from excessive environmental change and self-inflicted stress: the society of the only remaining homo species who might instead be remembered as fossils next to chairs, pavements, and electronics.
